# Investigation on Anthrax in Bangladesh during the Outbreaks of 2011 and Definition of the Epidemiological Correlations

**DOI:** 10.3390/pathogens10040481

**Published:** 2021-04-15

**Authors:** Domenico Galante, Viviana Manzulli, Luigina Serrecchia, Pietro Di Taranto, Martin Hugh-Jones, M. Jahangir Hossain, Valeria Rondinone, Dora Cipolletta, Lorenzo Pace, Michela Iatarola, Francesco Tolve, Angela Aceti, Elena Poppa, Antonio Fasanella

**Affiliations:** 1 Istituto Zooprofilattico Sperimentale of Puglia and Basilicata, Anthrax Reference Institute of Italy, 71121 Foggia, Italy; domenico.galante@izspb.it (D.G.); luigina.serrecchia@izspb.it (L.S.); valeria.rondinone@izspb.it (V.R.); dora.cipolletta@izspb.it (D.C.); lorenzo.pace@izspb.it (L.P.); michela.iatarola@izspb.it (M.I.); francesco.tolve@izspb.it (F.T.); angela.aceti@izspb.it (A.A.); elena.poppa@izspb.it (E.P.); antonio.fasanella@izspb.it (A.F.); 2 Servizio Igiene degli Allevamenti e delle Produzioni Zootecniche—Asl 02 Abruzzo Lanciano—Vasto-Chieti, 66054 Vasto, Italy; pietro.ditaranto@asl2abruzzo.it; 3 Department of Environmental Sciences, Louisiana State University, Baton Rouge, LA 70803-5705, USA; mhughj1@lsu.edu; 4 International International Centre for Diarrheal Disease Research, Programme on Infectious Diseases & Vaccine Sciences, Health System & Infectious Disease Division, Bangladesh (ICDDR,B), 1212 Dhaka, Bangladesh; jhossain@mrc.gm; 5 Medical Research Council Unit The Gambia at the London School of Hygiene and Tropical Medicine, 273 Banjul, The Gambia

**Keywords:** *Bacillus anthracis*, Bangladesh, epidemiology, MLVA, food contamination

## Abstract

In 2011, in Bangladesh, 11 anthrax outbreaks occurred in six districts of the country. Different types of samples were collected from May to September in the six districts where anthrax had occurred in order to detect and type *Bacillus anthracis (B. anthracis)* strains. Anthrax was detected in 46.6% of the samples analysed, in particular in soils, but also in bone samples, water, animal feed, and rumen ingesta of dead animals. Canonical single nucleotide polymorphisms (CanSNPs) analysis showed that all the isolates belonged to the major lineage A, sublineage A.Br.001/002 of China and Southeast Asia while the multi-locus variable number of tandem repeats (VNTRs) analysis (MLVA) with 15 VNTRs demonstrated the presence of five genotypes, of which two resulted to be new genotypes. The single nucleotide repeats (SNRs) analysis showed 13 SNR types; nevertheless, due to its higher discriminatory power, the presence of two isolates with different SNR-type polymorphisms was detected within two MLVA genotypes. This study assumes that soil is not the only reason for the spread of the disease in Bangladesh; contaminated feed and water can also play an important role in the epidemiology of anthrax. Possible explanations for these epidemiological relationships are discussed.

## 1. Introduction

Anthrax is a bacterial disease caused by the spore-forming *Bacillus anthracis,* affecting humans and animals. The disease is present in many countries in the world; however, the highest prevalence of anthrax cases is mainly recorded in tropical and subtropical areas [1]. In the last years, anthrax occurred in Turkey [2], Greece [3], Sweden [4], USA [5], Australia [6], Africa [7], Asia [8], Italy [9], Albania [10] and many other places in Europe. In Bangladesh, anthrax (popularly known as “Torka”) was periodically reported both in animals and humans between 1949 and 1986 [11,12]. The Department of Livestock (DLS) registered 437 animal cases in 2008 and 449 animal cases in 2009. In 2010, in Bangladesh anthrax cases involving animals and humans accounted for 104 and 607, respectively [13]. In this country, the occurrence of anthrax outbreaks depends on several conditions. First of all, anthrax outbreaks usually occur during the monsoon season, the period from April to November [14]. These events are due to favourable environmental conditions such as soil, pH, Ca^2+^ content, moisture, soil type, high ambient temperature, and rainfall, which are positively correlated with the persistence of anthrax spores in the environment and subsequent outbreaks [15,16,17]. Moreover, many people have no knowledge of the proper disposal process of carcasses, which are usually abandoned in open fields and in nearby river water [14]. In this situation, animals can be infected grazing in contaminated pastures with anthrax spores. In addition, cattle feed, often composed of animal bones contaminated with anthrax spores, can be considered an important source of infection [13]. As regards human cases, people contract anthrax from slaughtering, butchering, handling, and eating the meat of affected animals or due to contact with contaminated animal products, e.g., wool, meat, blood, hides. In addition, after the slaughtering of sick animals, meat is often sold at low cost to community members [13]. Furthermore, the Livestock Research Institute (LRI) is the only producer of livestock anthrax vaccine in Bangladesh, and it produces an average of 3.9 million doses of anthrax vaccines per year. The number of doses produced per year is far from providing a high livestock anthrax vaccination coverage because the total cattle population is about 23 million [18]. 

Under these circumstances, in 2011, eleven anthrax outbreaks occurred in six districts (Pabna, Sirajganj, Bogra, Faridpur, Meherpur, and Tangail Districts). These outbreaks included 122 humans suspected cases with two human deaths. The two deceased persons, a 70-year-old male from Bogra and a 40-year-old male from Tangail, had symptoms of suspected cutaneous and gastrointestinal anthrax [19]. As regards animals, 1668 cases were diagnosed, and 173 deaths were registered (Table 1) [18]. Given this situation, a collaborative investigation team from International Centre for Diarrhoeal Disease Research, Bangladesh (ICDDR,B) investigated the possible sources of contamination causing the outbreaks and collected samples from suspected sites (pasture lands, cattle sheds, slaughterhouses, and burial sites). Our work was focused on the isolation of *B. anthracis* from environmental, feed, and animal samples collected in the districts where anthrax outbreaks occurred.

## 2. Results

### 2.1. B. anthracis Detection by PCR

*B. anthracis* was isolated from 28/60 samples (46.6%). In particular those resulted positive were 15 out of 36 soil samples (41.6%), six out of seven bone samples (85.7%), one out of three water samples (33.3%), 3 out of 10 feed samples (30%) and three out of three rumen ingesta samples (100%). On the contrary, the meat sample collected in formalin was negative. 

### 2.2. CanSNPs, MLVA, and SNR Analyses

The canonical SNP analysis revealed that all the isolates belonged to the major lineage A, sublineage A. Br. 001/002, according to the worldwide distribution of *B. anthracis* clonal lineages [20]. This result has confirmed the presence of the same lineage shown in a previous investigation in Bangladesh [13]. 

**Figure 1:**
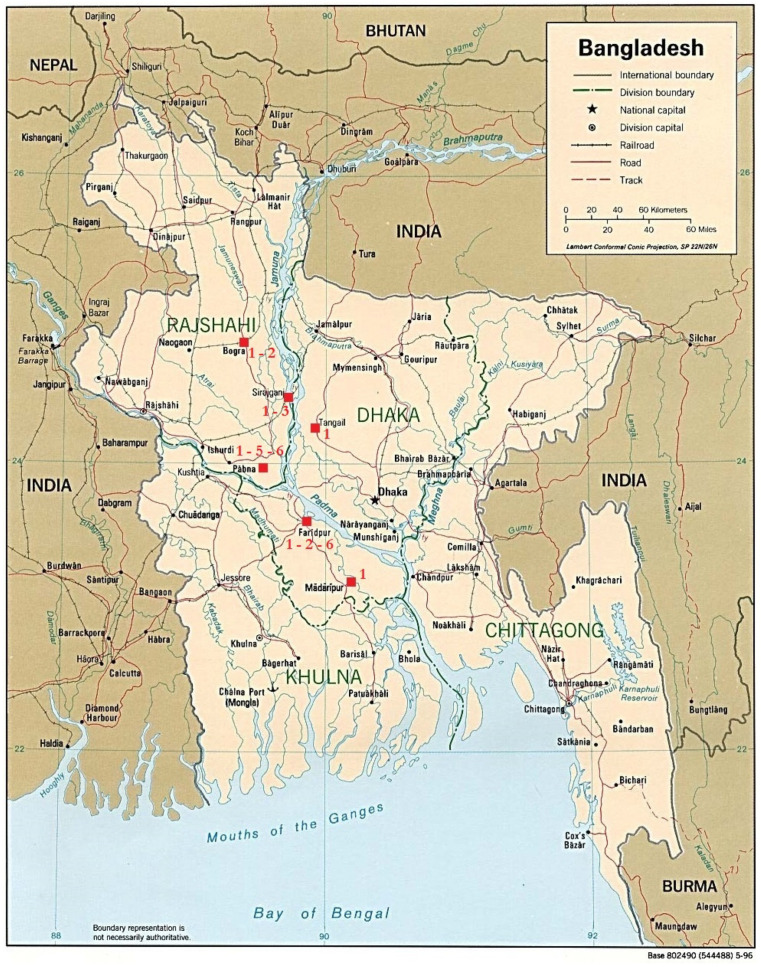
Distribution of the detected genotypes in the different districts of Bangladesh. 1: Gt_1/Ban; 2: Gt_2/Ban; 3: Gt_3/Ban; 5: Gt_5/Ban; 6: Gt_6/Ban. Image modified from “Map of Bangladesh”—“World of Maps”—Public Domain (Map of Bangladesh (Political Map): Worldofmaps.net—online Maps and Travel Information, Available at: https://www.worldofmaps.net/en/asia/maps-of-bangladesh/map-of-bangladesh-political-map.htm, accessed 14 April 2021).

The MLVA detecting VNTRs in 15 loci showed the presence of five genotypes (Figure 1; Table 2). Two of them (GT/KamBel and GT/ChU) correspond to the same genotypes previously identified in 2010 [13]. GT_2/Ban was already found out in the area of Sirajganj and Tangail [21]. The other two are novel genotypes labelled as GT_5/Ban and GT_6/Ban. 

GT1/Ban (or GT/KamBel) genotype was the most prevalent; in fact, it was present in all Upazilas except in Ullapara. It was isolated from 18 of 28 positive samples (64.3%), in particular, from rumen ingesta, bone, feed, and soil samples. 

GT3/Ban (or GT/ChU) genotype was detected from 4 of 28 positive samples (14.3%) samples collected in Sirajganj District. Samples consisted of water, bone, and soil.

GT_2/Ban genotype was isolated from 4 of 28 positive samples (14.3%) collected in Dhunat and Faridpur Upazilas, in particular, from bone and soil samples.

One bone sample of 28 positive samples (3.6%) collected in Santhia Upazila was contaminated with GT_5/Ban genotype. 

GT_6/Ban genotype was present in Faridpur and Santhia Upazilas. It was isolated from 2 out of 28 positive samples (7.14%), in particular, from feed and soil samples.

One feed sample, collected in Faridpur Upazila showed the coexistence of two genotypes (GT/KamBel and GT_6/Ban).

The SNR analysis showed the presence of 13 SNR types (SubGt-1, SubGt-1_1, SubGt-1_2, SubGt-1_3, SubGt-1_4, SubGt-1_5, SubGt-1_6, SubGt-1_7, SubGt-5_1, SubGt-5_2, SubGt-6_1, SubGt-6_2, SubGt-9). 

Eight different SNR-type polymorphisms (SubGt-1, SubGt-1_1, SubGt-1_2, SubGt-1_3, SubGt-1_4, SubGt-1_5, SubGt-1_6, SubGt-1_7) were detected within the MLVA genotype Gt/KamBel (or GT1/Ban). SubGt-1 was also detected within the MLVA genotype Gt_6/Ban.

Two different SNR-type polymorphisms (SubGt-6_1, SubGt-6_2) were detected within Gt/ChU (or GT3/Ban).

Gt_2/Ban presented two different SNR-type polymorphisms (SubGt-5_1, SubGt-5_2).

Two isolates showed novel SNR-type polymorphisms (SubGt-9) detected within the Gt_5/Ban and Gt_6/Ban. (see Table 3 for details).

## 3. Discussion

Results emerging from our study revealed the presence of five *B. anthracis* genotypes in the analysed samples; among these, GT5/Ban and GT6/Ban were identified as novel genotypes not found in previous studies [13,21]. GT1/Ban showed the highest percentage because it was widely expressed in all the sites. 

By biomolecular assays, 13 new sub-genotypes were found. Some of them were present in more than one genotype. For example, subgenotype-1 (subGt_1) was present within GT1/Ban but unexpectedly was also identified within GT6/Ban (Table 2). 

All the genotypes identified in this study were detected in samples from Rajshahi Division. Rajshahi is considered an important commercial area concerning the livestock market. We cannot exclude that during the journey through different areas of Bangladesh, animals might consume contaminated feed, developing anthrax before the arrival in the commercial area of Rajshahi. For these reasons, Rajshahi could be considered a focal point where different anthrax genotypes can mix. All these hypotheses are enforced by the presence of the same genotype (GT6/Ban) in a soil sample from Santhia (Rajshahi Division) and in a feed sample from Faridpur (Dhaka Division). The same situation occurs when analysing the GT3/Ban. This genotype was present in soil and water samples, both probed from Ullapara, but also in two bone samples from Sirajganj (Rajshahi Division) that is located about 30 km from Ullapara. 

Water and soil can be considered a source of contamination for animals during pasture, and consequently, anthrax transmission in livestock probably occurs during the consumption of contaminated homemade feed grown and water. Transmission via feed was already supposed in previous studies by interviewing farmers during outbreaks and asking them how livestock was fed [13]. 

Moreover, in contrast to previous studies, in which it was quite probable that anthrax transmission could not be linked to the food because feed samples resulted negative to *B. anthracis* [21], in this research, 3 out of 10 feed samples (30%) resulted positive to *B. anthracis*. 

It is presumable that animal infection can occur during the transport of animals from the wholesalers to the farms (grazing in contaminated pastures) or during the ingestion of homemade feed containing hay grown on anthrax contaminated soils. 

Another aspect to consider is that in Bangladesh, harmful feeds are used, named bone meals, which are also used for cattle. These feeds are prohibited in many countries because of their potentially dangerous effects on animal and human health. Bangladesh lacks the mechanism to test imported or locally produced animal products to determine if they carry any harmful chemicals, residues, or biological agents that pose serious threats to human and animal health. Feed contamination can occur during the process of production of bone meals with anthrax infected bones since viable spores can remain in feed or in the equipment to produce it [22]. Bones usually come from different villages and are mixed so that *B. anthracis* strains can be variable both in genotype and in numbers. In fact, by analysing one sample, it was surprising that two different genotypes (GT1/Ban and GT6/Ban) were present in a single feed sample. 

A mixed-genotype feed sample, as found here in the Faridpur feed samples, is absolutely typical of contaminated bone meal. It results in different strains deriving from anthrax dead animals from different farms. The hypothesis of contaminated feed is enforced by the presence of *B. anthracis* within three out of three rumen ingesta samples.

If feed is one of the causes for the spread of anthrax, soil presently remains the main source of infection. In fact, 15 out of 36 analysed soil samples confirmed the presence of *B. anthracis.* This can be explained by the fact that in Bangladesh the incorrect removal of infected carcasses results in high spore dissemination in the environment, increasing considerably the trend of this disease. Thus, the correct removal of infected carcasses and the improved control of food, with certifications of no anthrax spore contamination is crucial in order to prevent and control anthrax in Bangladesh.

This study confirmed that livestock feed is an important tool for the transmission of *B. anthracis* and that contaminated soils are not the only reason for the spread of the disease. The great abundance of analysed samples in different areas and sites of sampling increased the quantity and quality of data obtained in this study. Ultimately, the collection and the analyses of different matrix samples, such as feed, bones, rumen ingesta, water, and soil were useful to define the correlation between the different anthrax outbreaks, allowing to obtain relevant information about the epidemiology of *B. anthracis* in Bangladesh.

## 4. Materials and Methods

### 4.1. Sampling

The investigative team from the ICDDR,B collected 60 samples in different areas of Bangladesh and sent them to the Anthrax Reference Institute of Italy, Foggia. The samples were collected from May to September 2011. The investigation was conducted in sites where animal and human anthrax cases were registered. Precisely, nine samples were collected in Faridpur Upazila of Faridpur District, three samples in Ghatail Upazila of Tangail District, eight samples in Dhunat Upazila of Bogra District, 26 samples in Shahjadpur Upazila of Sirajganj District, five samples in Santhia Upazila of Pabna District, four samples in Ullapara Upazila of Sirajganj District and five samples in Gangni Upazila of Meherpur District. These consisted of 7 bone samples, 36 soil samples, 3 water samples, 10 grass silage samples (feed), 1 meat sample in formalin, and 3 rumen ingesta samples (Table 4). Grass silage samples, about 50 g each, were collected in farms where anthrax outbreaks had occurred. The soil samples, about 50 g each, were collected from suspected contaminated sites, in places where animals were slaughtered, buried, or died. 

### 4.2. Isolation

The ground anthrax *Bacillus* refined identification (GABRI) method was used to recover *B. anthracis* organisms from the soil samples [23]. This test, developed in the laboratories of the Anthrax Reference Institute of Italy, is able to isolate *B. anthracis* from contaminated soil with a detection limit of about 200 spores in 7.5 g soil (data not shown) or with a threshold value of 27 spores/g soil [23]. Briefly, 7.5 g soil was added to 22.5 mL washing buffer (sterile distilled water solution containing 0.5% Tween 20) and vortexed for 30 min. The solution was then centrifuged at 2000 rpm for 5 min and the supernatant was incubated at 64 °C for 20 min. After this incubation, 3 mL supernatant was mixed with 3 mL phosphomicin tryptose soya broth (PTSB), and 1 mL of the supernatant/PTSB was seeded onto a TSMP agar plate (trimethoprim, sulfamethoxazole, methanol, polymixine, and 5% sheep blood). The plates were incubated aerobically at 37 °C for 24 h. Suspected colonies on TSMP plates were morphologically observed and Gram staining was performed.

### 4.3. DNA Preparation and PCR 

Each *B. anthracis* suspect colony was streaked onto 5% sheep blood agar plates and then incubated at 37 °C for 24 h. After heat inactivation (98 °C for 20 min), microbial DNA was extracted using the DNAeasy Blood and Tissue kits (Qiagen, Hilden, Germany), following the protocol for Gram-positive bacteria. Specific PCR assays were used to confirm *B. anthracis* [24]. 

### 4.4. Canonical Single Nucleotide Polymorphism (CanSNP) Analysis

CanSNPs profiles, consisting of 13 TaqMan-Minor Groove Binding (MGB) allelic discrimination assays with oligonucleotides and probes, used as described by Van Ert et al. 2007 for each of 13 canonical SNPs [20]. Each 5 µL reaction contained 1× TaqMan Genotyping Master Mix (Thermo Fisher Scientific, Waltham, MA, USA), 250 nm of each probe, and 600 nm each of forward and reverse primers and 1.0 µL of ~1 ng template DNA. For all assays, thermal cycling parameters were as follows: 95 °C for 10 min, followed by 50 cycles at 95 °C for 15 s and 60 °C for 1 min. Endpoint fluorescent data were measured on the ABI 7900HT (Thermo Fisher Scientific, Waltham, USA). Canonical SNP profiles were compared to the 12 recognised worldwide sublineages and subgroups by TaqMan-Minor Groove Binding (MGB) allelic discrimination assays as described before [20]. 

### 4.5. The 15-Loci Multi-Locus Variable Number of Tandem Repeats (VNTRs) Analysis (MLVA) and Single Nucleotide Repeats (SNRs) Analysis

To obtain higher genetic differentiation in very closely related isolates, VNTRs loci were investigated, paired with SNRs loci that are molecular markers with extreme discriminatory power. This test is based on the use of 5′-fluorescent-labelled oligonucleotides, deprotected and desalted, specifically selected for the VNTRs and SNRs. The 15 specific primer pairs for the MLVA were selected as described by Van Ert et al. (2007) [20]. The four specific primer pairs for SNR reactions were selected according to Garofolo et al. (2010) [25]. The MLVA method provided seven PCR reactions divided into two Singleplex and five Multiplex, in a final volume of 15 µL. Each reaction mixture contained 1X PCR reaction buffer (Qiagen, Hilden, Germany); 3 mM MgCl_2_, 0.2mM for each dNTPs; 1UI Hot Star Plus Taq DNA polymerase (Qiagen, Hilden, Germany), and appropriate concentrations of each primer (Singleplex 1: vrrC1, 0.2 μM; Singleplex 2: vrrC2, 0.2 μM; Multiplex 1: vrrA, 0.2 μM; vrrB1, 0.2 μM and CG3, 0.4 μM; Multiplex 2: vrrB2, 0.25 μM; pXO2, 0.1 μM; pXO1, 0.3 μM; Multiplex 3: vntr12, 0.25 μM; vntr19, 0.2 μM; vntr35, 0.2 μM; Multiplex 4: vntr16, 0.25 μM; vntr23, 0.2 μM; Multiplex 5: vntr17, 0.1 μM; vntr32, 0.4 μM); and 2 μL of DNA. 

The PCR thermocycling program for two Singleplex and Multiplex 1 and 2 was the same, i.e., 95 °C for 5 min; 35 cycles at 94 °C for 30 s, at 60 °C for 30 s, and 72 °C for 30 s. The final step was at 72 °C for 5 min.

The amplification program for another Multiplex was the following: 95 °C for 5 min, 35 cycles to 94 °C for 30 s, 54 °C for 30 s, 72 °C for 45 s, and 72 °C for 5 min (Multiplex 3); 95 °C for 5 min, 35 cycles at 94 °C for 30 s, 56 °C for 45 s, 72 °C for 1 min, and 72 °C for 5 min (Multiplex 4); 95 °C for 5 min, 35 cycles at 94 °C for 30 s, 59 °C for 45 s, 72 °C for 1 min and 72 °C for 5 min (Multiplex 5).

Single nucleotide repeats (SNRs) analysis reveals the mononucleotide–nucleotide repeats, a type of variable number tandem repeat (VNTR) with a high rate of mutation (6.0 × 10^−4^ mutations per generation) present within the genome of *B. anthracis.*

This test allows the detection of the sub-genotype of our isolates within the same genotype previously identified with MLVA. In this study, we applied the modified SNR technique described by Kenefic et al. (2008) [26]. For the identification of the four SNRs, one Multiplex and one Singleplex PCR reactions were set up in a final volume of 12.5 μL, which contained 1X PCR Buffer (Qiagen, Hilden, Germany), 3.5 mM MgCl_2_, 0.2 mM of each dNTPs, 1 IU Hot Star Taq Plus DNA polymerase (Qiagen, Hilden, Germany), 2 μL of DNA and appropriate concentrations of fluorescent-labelled forward and reverse primers (Multiplex 1: HM1 (CL33), HM13 (CL35), 0.2 μM; HM6 (CL12), 0.1 μL; Singleplex 1: HM2 (CL10), 0.2 μM). For both reactions, the PCR cycle provided the following amplification process: 95 °C for 5 min, 35 cycles at 94 °C for 30 s, 60 °C for 30 s, 72 °C for 30 s, and 72 °C for 5 min.

### 4.6. Automated Genotype Analysis

The MLVA PCR products were diluted 1:80 and subjected to capillary electrophoresis on ABI Prism 3130 Genetic Analyser (Thermo Fisher Scientific, Waltham, USA) with 0.25 µL GeneScan 1200 and sized by GeneMapper 4.0 (Thermo Fisher Scientific, Waltham, USA). Amplified SNR PCR products were diluted 1:80 and subjected to capillary electrophoresis on ABI Prism 3130 genetic analyser (Thermo Fisher Scientific, Waltham, USA) with 0.25 µL GeneScan 120 LIZ, and sized by GeneMapper 4.0 (Thermo Fisher Scientific, Waltham, USA). In all the analyses, the samples were processed in triplicate and the concordance of the results allowed the correct sizing of the fragments. Amplified SNR PCR products were diluted 1:80 and subjected to capillary electrophoresis on ABI Prism 3130 Genetic Analyser (Thermo Fisher Scientific, Waltham, USA) with 0.25 µL GeneScan 120 LIZ, and sized by Gene Mapper 4.0 (Thermo Fisher Scientific, Waltham, USA).

## Figures and Tables

**Table 1:** Species distribution of the estimated number of diagnosed cases and death cases in Bangladesh, 2011.

	Diagnosed Cases (%)	Deaths (CFR ^1^, %)
Cattle	1278 (76.6%)	165 (12.9%)
Buffaloes	83 (5.0%)	3 (3.6%)
Goats	270 (16.2%)	5 (1.9%)
Sheep	37 (2.2%)	0
Humans	122	2 (1.6%)

^1^ CFR= case fatality rate.

**Table 2:** Genotype based on a multi-locus variable number of tandem repeats (VNTRs) analysis (MLVA) with 15 VNTRs.

					*VNTRs Observed bp*																
Division	District	Upazila	Sample Type	Sample ID	vrra	vrrb1	vrrb2	vrrc1	vrrc2	cg3	pXO1	pXO2	vntr12	vntr16	vntr17	vntr19	vntr23	vntr32	vntr35	MLVA 15 Gt	
DHAKA	*Faridpur*	*Faridpur*	*Bone*	F26/1-B	306	223	154	584	522	153	121	133	110	135	381	90	182	378	116	Gt_2/Ban	[21]
				F25-B	306	223	154	584	522	153	127	133	110	135	381	90	182	378	116	Gt_1/Ban	Gt/KamBel [13]
			*Feed*	F25/1-F	306	223	154	584	522	153	127	133	110	135	381	90	182	378	116	Gt_1/Ban	Gt/KamBel [13]
				F25/1-F	306	223	154	584	522	153	127	135	110	135	390	90	182	378	116	Gt_6/Ban	new
			*Soil*	F25-S	306	223	154	584	522	153	127	133	110	135	381	90	182	378	116	Gt_1/Ban	Gt/KamBel [13]
	*Tangail*	*Ghatail*	*Soil*	G001-S	306	223	154	584	522	153	127	133	110	135	381	90	182	378	116	Gt_1/Ban	Gt/KamBel [13]
			*Slaughter soil*	G001-SS	306	223	154	584	522	153	127	133	110	135	381	90	182	378	116	Gt_1/Ban	Gt/KamBel [13]
			*Rumen*	G001-R	306	223	154	584	522	153	127	133	110	135	381	90	182	378	116	Gt_1/Ban	Gt/KamBel [13]
KHULNA	*Meherpur*	*Gangni*	*Soil*	M002/1-S	306	223	154	584	522	153	127	133	110	135	381	90	182	378	116	Gt_1/Ban	Gt/KamBel [13]
				M002/2-S	306	223	154	584	522	153	127	133	110	135	381	90	182	378	116	Gt_1/Ban	Gt/KamBel [13]
				M002/3-S	306	223	154	584	522	153	127	133	110	135	381	90	182	378	116	Gt_1/Ban	Gt/KamBel [13]
RAJSHAHI	*Bogra*	*Dhunat*	*Slaughter soil*	D5003-SS	306	223	154	584	522	153	121	133	110	135	381	90	182	378	116	Gt_2/Ban	[21]
				D5006-SS	306	223	154	584	522	153	121	133	110	135	381	90	182	378	116	Gt_2/Ban	[21]
			*Soil*	D5007-S	306	223	154	584	522	153	121	133	110	135	381	90	182	378	116	Gt_2/Ban	[21]
			*Rumen*	D5007-R	306	223	154	584	522	153	127	133	110	135	381	90	182	378	116	Gt_1/Ban	Gt/KamBel [13]
	*Pabna*	*Santhia*	*Bone*	PSC006-B	306	223	154	538	522	153	130	137	110	135	381	90	182	563	122	Gt_5/Ban	new
			*Feed*	PSC006-F	306	223	154	584	522	153	127	133	110	135	381	90	182	378	116	Gt_1/Ban	Gt/KamBel [13]
			*Soil*	PSC006-S	306	223	154	538	522	153	130	137	110	135	381	90	182	563	122	Gt_6/Ban	new
	*Sirajganj*	*Shahjadpur*	*Bone*	S05-B	295	223	154	584	522	153	127	133	110	135	381	90	182	563	116	Gt_3/Ban	Gt/ChU [13]
				S13-B	295	223	154	584	522	153	127	133	110	135	381	90	182	563	116	Gt_3/Ban	Gt/ChU [13]
				S01-B	306	223	154	584	522	153	127	133	110	135	381	90	182	378	116	Gt_1/Ban	Gt/KamBel [13]
			*Feed*	S01-F	306	223	154	584	522	153	127	133	110	135	381	90	182	378	116	Gt_1/Ban	Gt/KamBel [13]
			*Rumen*	S01-R	306	223	154	584	522	153	127	133	110	135	381	90	182	378	116	Gt_1/Ban	Gt/KamBel [13]
			*Soil*	S01-S	306	223	154	584	522	153	127	133	110	135	381	90	182	378	116	Gt_1/Ban	Gt/KamBel [13]
				S01_S22-S	306	223	154	584	522	153	127	133	110	135	381	90	182	378	116	Gt_1/Ban	Gt/KamBel [13]
				S01_S12-S	306	223	154	584	522	153	127	133	110	135	381	90	182	378	116	Gt_1/Ban	Gt/KamBel [13]
				S11-S	306	223	154	584	522	153	127	133	110	135	381	90	182	378	116	Gt_1/Ban	Gt/KamBel [13]
		*Ullahpara*	*Soil*	U05-S	295	223	154	584	522	153	127	133	110	135	381	90	182	563	116	Gt_3/Ban	Gt/ChU [13]
			*Water*	U10-W	295	223	154	584	522	153	127	133	110	135	381	90	182	563	116	Gt_3/Ban	Gt/ChU [13]

**Table 3:** Sub-genotype based on single nucleotide repeats (SNRs) analysis.

MLVA 15 Gt	SNR Considered				SNR_4 subGt
	HM1 CL33/pXO2	HM2 CL10/Chrom	HM6 CL12/Chrom	HM13 CL35/pXO2	
*Gt_1/Ban or Gt/KamBel*	82	111	90	118	*subGt_1*
	82	109	90	118	*subGt_1_1*
	83	112	90	118	*subGt_1_2*
	85	106	92	119	*subGt_1_3*
	82	109	91	119	*subGt_1_4*
	80	111	89	118	*subGt_1_5*
	82	109	90	119	*subGt_1_6*
	83	109	91	119	*subGt_1_7*
*Gt_2/Ban*	82	109	90	119	*subGt_5_1*
	83	109	91	118	*subGt_5_2*
*Gt_3/Ban or Gt/ChU*	79	108	91	118	*subGt_6_1*
	85	106	92	119	*subGt_6_2*
*Gt_5/Ban*	83	109	91	118	*subGt_9*
*Gt_6/Ban*	82	111	90	118	*subGt_1*
	83	109	91	118	*subGt_9*

**Table 4:** Sample types and geographical distribution.

Division	District	Upazila	Bone	Rumen Ingesta	Soil	Feed	Water	Meat in Formaline
DHAKA	*Faridpur*	*Faridpur*	2	-	4	3	-	-
	*Tangail*	*Gathail*	-	1	2	-	-	-
KHULNA	*Meherpur*	*Gangni*	-	-	4	-	-	1
RAJSHAHI	*Bogra*	*Dhunat*	-	1	7	-	-	-
	*Pabna*	*Santhia*	2	-	2	1	-	-
	*Sirajganj*	*Shahjadpur*	3	1	15	5	2	-
		*Ullapara*	-	-	2	1	1	-
